# Transforming identity through participation in music and theatre: exploring narratives of people with mental health problems

**DOI:** 10.1080/17482631.2017.1379339

**Published:** 2017-09-14

**Authors:** Kristin Berre Ørjasæter, Theodore Stickley, Marianne Hedlund, Ottar Ness

**Affiliations:** ^a^ Faculty of Nursing and Health Sciences, Nord University, Bodø, Norway; ^b^ Faculty of Medicine and Health Sciences, Norwegian University of Science and Technology, Trondheim, Norway; ^c^ Faculty of Medicine and Health Sciences, University of Nottingham, Nottingham, UK; ^d^ Faculty of Social and Educational Science, Norwegian University of Science and Technology, Trondheim, Norway; ^e^ Department of Health, Social and Welfare Studies, University College of Southeast, Drammen, Norway

**Keywords:** Arts, identity, identities, mental health, music, narrative, recovery, theatre, workshop

## Abstract

**Background:** There is a growing understanding that mental health problems and prolonged contact with mental healthcare systems can affect people’s identities. Working with identity is an important element in mental health recovery.

**Purpose:** In this article, we explore the significance of participation in a music and theatre workshop in terms of people`s experiences of identity.

**Design and methods:** This is a qualitative study based on a hermeneutical phenomenological epistemology. Data were collected from in-depth interviews with 11 participants at a music and theater workshop, analysed through a narrative analysis and presented in an ideographical “long” narrative form. The music and theater workshop is not overtly therapeutic although the activity takes place in a Norwegian mental health hospital for adults living with long-term mental health problems.

**Results:** We identified three crosscutting themes: (1) *becoming a whole person,* (2) *being allowed to hold multiple identities* and (3) *exploring diverse perspectives*.

**Conclusion:** Findings show that participation in the music and theatre workshop transformed the participants’ experiences of identity on two levels: individually and collectively. The participants developed a broader picture of themselves through their creative work with others. When they developed new identities, the narratives of themselves expanded.

## Introduction

In her 2000 book, *From Psychiatric Patient to Citizen*, Liz Sayce envisaged a collective shift in identity for those who have been diagnosed with a mental illness. In 2016, she released a new edition and questioned, “to what extent have the rights of citizenship progressed in the 16 years gap between the books?” (Sayce, 2016). Whilst European countries may have seen an increase in media campaigns, and governments are more willing to discuss mental health, what is less clear is what progress has been made in the collective identity of those with a diagnosis of mental illness.

Throughout history, language regarding mental illness has described a collective and largely negative identity: lunatics, the insane, the mad, and so on. What each of the nouns has in common is its pejorative nature. Historically, the so-called mad were sent to an asylum, set apart from mainstream society, and the identity of the “outcast” or the “other” was established (Davidson, Strauss, & Rakfeldt, 2010). In many countries, the large asylums have been closed, but the stigma of people with mental health problems remains entrenched (Sayce, 2016). They can experience what Goffman (1992) called “spoiled identities.” Goffman claimed that people with a spoiled identity feel inferior and discredited and almost inevitably become so. As Goffman pointed out, identity is not an unaltered concept, but socially defined. Consequently, identity is neither permanent nor static, but something that is dynamic and constantly under development (Erikson, 1968). For people who experience mental distress, identity issues stretch back to the formative years of life when self-worth was established (Rogers, 1967). Any change in a person’s identity will inevitably take time, involve a processes of negotiation and reference to anchor points.

In this article, we employ a narrative inquiry methodology that has the concept of identity at its core. By analysing individual narratives of people who have lived with mental distress and resultant diagnosis, we seek to discover and understand people’s identity claims and if and how participating in arts activities leads to new identity claims. Through narrative discourse, identity is “accomplished, disputed, ascribed, resisted, managed and negotiated” (Benwell & Stokoe, 2006, p. 4). This approach to research elicits individual stories of people’s lives and their experiences and understanding of the meaning they ascribe to those stories; it is within this meaning that people locate their own identity and make sense of their lives (Denzin, 2000). People may “position” their identity in relation to greater social narratives or discourses (Phillips & Hardy, 2002) or master narratives (Nelson, 2001) to help make sense of their experiences. Identities are negotiated and renegotiated with and in relation to others in society (Goffman, 1992; Nelson, 2001; Rogers, 1967). As there is a need to negotiate identity, people also may renegotiate negative identities (Riddell & Watson, 2014). In modernity, according to Bauman (2001, p. 129), people are no longer “having an identity” as they are “belonging to their” fate or historical categorization of identity. Instead, Bauman (2001) argued that sociopolitical, cultural, professional, religious and sexual identities are undergoing a process of continual transformation. Accordingly, identities become more fluid in modern times, meaning that identity is not a settled category without alternatives, but something more blurry and interchangeable with social contexts. Such understandings are relevant to this study.

There is scant evidence in the literature of narrative accounts that describe how people may negotiate their identities whilst participating in arts activities in hospitals. This study can be a complement to previous studies (Sagan, 2014, 2015; Spandler, Secker, Kent, Hacking, & Shenton, 2007; Stickley, 2010; Stickley & Duncan, 2007; Stickley & Eades, 2013; Swan, 2013; Van Lith, Fenner, & Schofield, 2009), which have illustrated from a first-person perspective that participation in community arts activities mainly conducted in their local communities has the potential to transform people’s identities. To our knowledge, few international studies (Sapouna & Pamer, 2014) and no Scandinavian studies have focused on participation in arts activities in hospitals in relation to mental health recovery and identity. This article provides a deeper understanding through long-ideographic data from people with long-term mental health problems participating in arts activities in a Norwegian mental health hospital. The research question for this study is: “What meaning does participation in a music and theatre workshop have for people’s experience of their own identity?”

### The music and theatre workshop as a research context

The research context was a music and theatre workshop (MTW) that was a leisure activity for people hospitalized in a Norwegian mental health hospital; the workshops were not therefore overtly therapeutic. Some participants used this as a work-training facility in collaboration with the Norwegian Labour and Welfare Service. Since its inception in 2003, approximately 60 people with mental health problems have participated in the MTW. The workshop uses a separate building located at the mental health hospital. A theatre director is employed full-time to run the MTW, which is open for participation regardless of diagnosis or previous experience with the arts.

Based on the participants’ interests and skills, the MTW provides activities in various art forms. The participants collaborate with a theatre director and professional actors and musicians, individually and in groups. A central working principle in the MTW is to make participants cocreators, and as such, they are involved in all aspects of the production process: script writing, singing, playing instruments, creating costumes, acting, providing technical support and so on. People bring their own poems, written drafts, and diary notes to the theatre director. Some of the participants are encouraged to transform their material into dialogues or songs. In working with professional musicians, participants bring a whole or part of a song and create a melody together with the musicians. They can also try different musical interpretations of the lyrics or just improvize with different instruments and from that create music.

The MTW has weekly rehearsals. In these rehearsals, the participants read and portray characters based on their written scripts. Through reading scripts in rehearsals, both participants and the theatre director get ideas for how new and old scripts could function together in a play. Usually, a bigger music and theatre production is developed once a year as well as the small-scale performances that takes place. Linked to the performances, the MTW collaborates with the Leisure Centre staff at the hospital, who facilitate technical, practical and personal support; stage work, transport and catering; and have informal conversations with the participants.

## Methods

For this article, we drew on data from a broader qualitative research study that explored recovery processes among people with long-term mental health problems. Situated within a hermeneutical phenomenological epistemological perspective (Van Manen, 1997, 2014), we used a narrative inquiry (Riessman, 2008) to explore and illuminate the phenomenon of participation. Van Manen (1997) argued that the meaning of any phenomenon is complex and manifold. For this reason, it is important to study the phenomenon in the complexity in which it exists. Following Finlay (2002, 2012) and Malterud (2011), we approached the data with reflexivity, exploring how our intentions and preconceptions as researchers influenced the study.

### Ethics approval

The Regional Committee for Medical and Health Research Ethics in Norway (REK, 2015/476) approved this study. Participation was voluntary and the participants were informed that they could withdraw their consent at any time without suffering any consequences. The Regional Committee considered the participants as a vulnerable group and a special consideration for the group’s interest during the research process was required (National Commitee for Research Ethics in the Social Sciences and the Humanities, 2006). In the study, a psychiatric specialist from an outpatient clinic near the music and theatre workshop was available to participants who wanted to speak to somebody after participating in the interviews. In the information sheet about the study, the specialist’s name, phone number and email address were given to all participants.

### Participants

In all, 11 participants from the MTW were recruited. Participants had to meet the following inclusion criteria: experiences of long-term mental health problems and current or former participation in the MTW. The theatre director distributed posters for participation to all 14 current participants and six of the former participants of the MTW. Those who were willing to participate could contact the first author via email, phone or pre-addressed envelope and were invited to a meeting about the study. All participants gave oral and written consent before their interview.

Seven women and four men agreed to participate, ranging in age from 22 to 48 years. They participated in the MTW from 9 months to 10 years. Their contact with the mental-health-care system ranged from 3 to almost 30 years. Health information was provided by the participants themselves, including their self-reported diagnosis. All but one participant still received various services related to their mental health problems from the municipality and/or the hospital.

### Data collection

The first author collected data by conducting 11 qualitative, conversational in-depth interviews (Kvale & Brinkmann, 2009; Patton, 2015). The qualitative interviews aimed to obtain descriptions of the interviewees’ lifeworld to interpret the described phenomenon (Kvale & Brinkmann, 2009). The interviews were deliberately conducted in conversational form, combined with an interview guide. The interviews were approached using open-ended questions (Patton, 2015), and the participants were invited to tell their stories with a minimum of interruptions (Bell, 1988), beginning with, “Could you tell me something about yourself, who you are? Can you tell me about your participation in the MTW?”

In the process of becoming familiar with the MTW, the first author observed the location and the group at rehearsals and performances. Then the first author conducted all interviews at locations selected by the participants. The majority chose to be interviewed in an office located in the same building where the MTW had its sessions. Three participants chose other locations: in their home, in a district psychiatric centre or at a forensic hospital. The participants were interviewed once. The interviews ranged from 46 to 138 minutes and were carried out between June and October 2015. All of the interviews were audio recorded and transcribed verbatim. To ensure anonymity, the participants’ names have been altered.

### Data analysis

Narrative analysis refers to a family of approaches to interpret texts that have a storied form in common (Riessman, 2005). Our analysis was inspired by Riessman’s (1993, 2008) narrative thematic analysis. Here, the exclusive focus of the analysis was on the content of the analysed transcripts. Focusing on the stories told, we examined the lives of the participants, honouring the principle of lived experience as a source of knowledge and understanding when analysing data (Clandinin, Steeves, & Caine, 2013).

The authors’ backgrounds have influenced the analysis and it is important for us to acknowledge that other researchers may have interpreted the data differently. However, the contributions of the four authors, which together reflect broad clinical and research experience in health science, may have increased rigour. The first author is trained as a clinical social worker and family therapist, while the second author is a mental health nurse. Both had long experiences as therapists in the mental-health-care system before becoming involved in qualitative interdisciplinary research in the field of arts and health. The third author is a medical sociologist and professor of health science. The fourth author is trained as a family therapist and professor of counselling with extensive research experience in mental health recovery.

The first author mainly conducted the data analysis. NVivo 11 (Qualitative Solution and Research International, 2015) and MindManager 2017 (Corel Corporation, 2017) were utilized as tools for analysing the data. Shortly after completion of each interview, oral memos of initial impressions of both the interview setting and the interview content were audio recorded. Inspired by Finlay (2012), the following questions were asked: What was the interview about? What stories did the participants tell? How was the interview climate experienced in the conversation? How did the analytic process affect the questions? Each interview was transcribed verbatim shortly after completion of the interview (Riessman, 1993). To become familiar with the data and gain insight into what stories the participants told, interview transcripts were read through several times. Then the first author wrote reflexive memos (Finlay, 2002, 2012) based on listening to the audio recordings and reading and re-reading the transcripts. Based on the reflexive memos, the first author summarized the essence of each story told. These summaries of “essence” in the stories were compared with the essences of stories from the other participants. The first author found and visualized motifs to create a map (Corel Corporation, 2017) with the following themes: (1) becoming a person, (2) moving away from a narrow identity and (3) developing a creative identity. To ensure trustworthiness, the authors met to discuss themes to check for alternative interpretations (Finlay, 2011; Lincoln, Lynham, & Guba, 2011; Schwandt, Lincoln, & Guba, 2007). In addition, themes and narratives were presented and critically discussed in different ways: at an open meeting with the participants and people involved in the MTW, with the participants who owned the narratives, on a dialogue forum for practice-oriented research in Norway, and at the Culture, Health and Well-Being International Conference in the UK. Based upon the feedback from these meetings, the first author went back to the data and added to the analysis; some themes were modified. Three cross-cutting themes emerged (Riessman, 1993, 2008): (1) becoming a whole person, (2) being allowed to hold multiple identities and (3) exploring diverse perspectives. From the 11 narratives, we chose three that broadly illustrated each of the cross-cutting themes to become the focus of this article. Therefore, the narratives should be seen as illustrations of the cross-cutting themes. All authors contributed to the writing process to create an expanded understanding of what the cross-cutting themes and narratives could tell us. Throughout the writing process, the analysis continued to be developed (Van Manen, 1997).

### Methodological strengths and limitations

A strength of the study was the in-depth conversational style used in the interviews, which were sufficiently open to enable participants to share rich stories regarding their participation in the MTW. To present our findings, we have chosen descriptions close to the participants’ accounts. Instead of using quotations from all participants, we chose to illustrate our cross-cutting themes with long-idiographical narratives from three of the participants. By illustrating the themes with these rich narratives, we were able to show the complexity, tension and contradiction within the participants. However, this could limit the reader’s understanding of how the cross-cutting themes were observed in the other participants.

## Findings

In the analysis process, we identified commonality amongst participants’ accounts of their experiences of engagement with the MTW; each of these accounts is embedded within a personal history and context. We identified three cross-cutting themes: (1) becoming a whole person, (2) being allowed to hold multiple identities and (3) exploring diverse perspectives. We present the themes separately by giving a short presentation of the content of each theme, illustrating each theme with a narrative, and reflecting on the narrative by considering the findings in light of the research literature. It is important to note that the identity narratives of Mina, Nelly and Oliver were fragmented and disordered, not explicitly told within a temporal order. We have organized the different stories from each of the interviews in a structure that facilitates interpretation of meanings.

### Theme 1: becoming a whole person

Theme 1 shows the different trajectories the participants shared about their experiences of becoming a whole person through participation in music and theatre. The participants described a wide range of processes that were important to them, as they allowed them to get to know themselves better and reveal new aspects of themselves without “wearing a mask.” Through retelling some of Nelly’s narrative, we illustrate some of the elements of this theme.

### Nelly: the peeling of layers

I feel like someone from another planet that has been thrown down here. All my life, I have desperately tried to find my place, while keeping up a facade for everyone around me. It has been important to camouflage my inner chaos by appearing resourceful and competent. I have had an inside and an outside that were never in harmony. A lot of my energy has been spent trying to make my inner chaos invisible to others, but I wish I could move in harmony with how I feel, because when the gap between the inside and the outside gets too wide, my entire life falls apart and the road to admission to hospital is short!

When the MTW first started, I had spent a lot of time in mental healthcare, but never wanted to be associated with any of it, like the day care centres, any possible groups or outings like the organisation “Mental health” or the leisure centre at the hospital—none of it. I really wanted to participate in the MTW, but that meant possibly being identified by someone, and then they could say: “Oh, so she’s one of those mentally ill people, then.” But then, when I had completed my studies, I could not start working. After filling my days with studies, it was back to everyday life! The gap between inside and outside became too wide again and I was admitted for a longer period. I took a risk and decided to explore the MTW. The level of generosity and non-judgmental attitude I was met with at the MTW was unlike any other. I became a regular there, even though I was afraid that anyone could find out that this was a group for the mentally ill. I still don’t always tell people what kind of group it is when I invited them to come and see it. I am afraid of the prejudice, even though I feel there is great quality to the work we do here as amateurs.

I do not feel like mental healthcare ruins my identity, but in a way, it does. They keep defining me, and it becomes an enduring reality. Of course, some of my problems might fit the diagnoses, but it is still not all of me. It is what they see. That is why it is so important to have an arena like the MTW when admitted, especially when you experience such loss of identity after remaining there for a long time. If the mental healthcare system wants us to grow and become independent people with healthy identities, it should make room for us, for the entire person, not just for the part that is ill.

After several years as an actor, I accepted the challenge of writing texts for the MTW, even though I was afraid that what I wrote was silly or not as good as what the others wrote. At the same time, I was terrified of exposing myself. My texts were about difficulties I had experienced myself, and existential questions I had that made it very personal. But I cannot keep avoiding everything; I need to show a little bit of the inside, or it will catch up with me again. I am working on accepting how things are and how things have become, that I am who I am as a person. I just want to be able to be a human being, to feel complete and whole.

### Analysis

Nelly experienced a year-long struggle with her identity. During this period, she needed to find a place where she felt she could belong. A sense of belonging has been previously associated with participatory arts activities (Lagacé, Briand, Desrosiers, & Larivière, 2016; Stickley, 2010; Van Lith, Fenner, & Schofield, 2011). Her need for belonging included a need to feel psychologically contained as she was afraid that her inner chaos might be visible to others. By participating in the MTW, Nelly applied her energies to developing a new role in the group, which demonstrated her competence and resourcefulness, in spite of her vulnerabilities. The chaos Nelly experienced (she referred to it as the “gap” between her inner and outer self) could only be helped by others. Her experiences with mental health services, however, were that they focused upon her deficits and weaknesses; participating in the MTW meant that she could develop her strengths and abilities and additionally access help in a relatively nonstigmatising environment. As such, the MTW helped her to process her identity issues more than standard psychiatric treatment possibly could. The apparent success of her engagement with the MTW may be largely attributed to the fact that whilst she was encouraged to maximize her strengths and abilities, there was also room for her vulnerabilities. This was successfully facilitated by a nonjudgemental environment, which gave her the space to acknowledge and explore her “gap.” As a result of this work, the gap narrowed and she experienced herself as a more complete person.

Nelly’s participation in the MTW was not without challenges and not all her problems were solved. Nevertheless, through participatory arts she was given an opportunity to be defined as a creative person rather than as a person having mental health problems (Sagan, 2015; Spandler et al., 2007; Stickley & Eades, 2013). Because of the stigma associated with mental health problems, it is understandable that people are strongly reluctant to disclose and become labelled for fear of alienation (Riddell & Watson, 2014).

The fact that Nelly considered the performances to be of high quality made it easier for her to publicly perform in an MTW production even though it might be known that the group was solely for people with mental health problems. The psychological benefits of performing (higher self-esteem and confidence) outweighed the potential stigma.

Nelly told a story of a long and painful journey. Participation in the MTW did not immediately remove her challenges. In most areas of her life, things remained unchanged from before she started in the MTW. Her story showed that she is in a process of change; she made no claim of a transformed identity, and her identity remained fragmented. Nevertheless, Nelly’s narrative illustrates that her participation in the MTW has been crucial for her to initiate the process of identity re-integration on her journey to becoming a whole person (Van Lith et al. (2009). An important discovery for her has been that participatory arts can give her the space needed to allow her to be herself and to become herself.

### Theme 2: being allowed to hold multiple identities

Theme 2, being allowed to hold multiple identities (Erikson, 1968), presents the participants’ struggle of having more than one dominant identity in society. The participants experienced that mental health patienthood often became a dominant identity in their lives and barred them from holding multiple, healthy and positive identities. They described that any other (more positive) identity could be neglected, overlooked or forgotten by themselves or others. We illustrate some of these experiences with an excerpt from Oliver’s story.

### Oliver: reclaiming life

I had been asking to be admitted to the mental healthcare system for a long time. I broke a glass of water against my forehead when my MD yet again denied my request for admission. That turned into a police escort to the hospital. It was important, because my life was in fact hanging in the balance. I knew I would be dead without treatment.

Life in the mental healthcare system gradually became like being in prison. I kept being reminded that I only had a status as a patient, and the need to have a different role than just that of a patient began to emerge. Thus, it was liberating to be treated as an actor instead of a patient in the MTW. The fact that nobody used diagnoses on the patients was important. Many of us had been in and out of the mental healthcare system; in and out of the institution. Several among us had low self-esteem and institutionalized role-identities. As an actor, I am neither ill, nor healthy. Actors act, they don’t self-harm, right? I got to develop a role identity as an actor and a self-image as a human being, not as a patient! The healthy part of the humanity kicked in, and I could start taking care of myself again.

While I was an actor in the MTW, I was also a prominent person in politics. Being open in both contexts at the same time cost me a lot. After a while, even the MTW could not give me the sense of belonging I was looking for. Again, the need to redefine my life emerged. So, I had a potential for development within the framework of the MTW. Once I had filled that frame, a need for greater challenges outside those of the MTW emerged. I did so much creative work in society at large that I no longer associated myself with the mental healthcare system, and continuing as an actor in the MTW became gradually more difficult with regards to my reputation as a politician. The MTW had actually given me the tools I needed to handle a lot of things, especially within the creative field, work life and social settings.

### Analysis

Oliver’s narrative refers to a story of identity and of continuous development of identity at various stages of his life. The identity he sought and experienced as liberating in one stage of his life made him feel stigmatized and hampered his personal growth at another stage. This resulted in a form of crisis and an urge to redefine himself. Oliver has a need for multiple identities in the same phase of life, but struggles to accept the fluidity and inconsistencies sometimes experienced with the reality of multiple identities (Bauman, 2001).

Prior to being hospitalized, Oliver reached a point in his life where he was no longer able to take care of himself. He described a long battle to obtain the status as a patient and the extreme measures that he was forced to take to achieve admission. Although Oliver initially wanted to become a patient, he gradually came to see that status as being identified as metaphorically “straightjacketed.” He was robbed of his sense of freedom due to the many demands and restrictions of the mental-health-care system. Furthermore, he experienced how difficult it made maintaining his roles and activities outside of the system (Goffman, 1968a). Oliver’s need for help over a prolonged period caused an identity crisis for him and ultimately led to his becoming a “mental patient” and having to reluctantly accept an “illness identity” (Sayce, 2016). This enabled him to have respite from his identity as a politician. However, he soon needed to discard the illness identity and establish a more positive role. Participation in the MTW provided this new identity. Previous studies have also observed the phenomenon of transformation from “illness” to “artist” identities amongst participants of group-based arts activity workshops (Sagan, 2014, 2015; Spandler et al., 2007; Stickley, 2010; Stickley & Duncan, 2007; Stickley & Eades, 2013; Van Lith et al., 2009).

Apparently, being perceived and subsequently treated as an actor in the MTW enabled Oliver to assume greater responsibilities, be more independent and develop new coping mechanisms, which in turn enabled him to start taking better care of himself. After a few years in the MTW, however, Oliver felt the need for more creative challenges outside of the hospital context. It appears that he began to feel his participation in the MTW was standing in the way of his becoming a creative and fully functioning citizen. Again, Oliver described the story of struggling with re-integrating his identity. Personal identities can never be separated from the societal and stigmatized identities associated with mental patients (Bauman, 2001).

Despite Oliver’s desire to combine his identities as an actor and a serious politician, he experienced these as incompatible. Although it seemed he could manage the transition from one identity to another, it appears that he believed he must choose one or the other. In the period when he spent most of his time outside the mental-health-care system, he felt the need to separate himself from the MTW in order to be taken seriously by others and not to chance being stigmatized as an actor with mental health problems. However, it is possible to see the liberation as a natural process in his recovery journey. Eventually, he sought more confirmation from his community outside the institution than from the hospital. Therefore, it may be natural to re-negotiate his identity and represent himself as a multicreative person, without simultaneously connecting this to his identity as being mentally ill.

### Theme 3: exploring diverse perspectives

Theme 3 illustrates how participation in the MTW contributes to participants’ exploration of diverse perspectives. In this process, the creative product of the group becomes more important than a single actor’s performance. In terms of creative processes, the group creates a performance piece based upon their own scripts. They are directed to act out a situation in different ways to figure out the best way to reach the audience. It is through this creative process that participants challenge themselves creatively and discover new aspects of themselves. We illustrate this theme by presenting some of Mina’s narrative.

### Mina: from no one to someone

The way I see things is probably very different from how many others see themselves, especially when it comes to my experience of being met by this mental healthcare system and not feeling like I have been reduced to just being a patient by it. I am, of course, a patient from time to time; this I can agree with, but I do not describe myself as ill, and I hate being labelled as such. I rather think of myself as having a low level of functionality.

In fact, I was initially sceptical towards participating in the MTW; I figured my poetry and music skills were better left at home and lacked the quality needed in this arena. I had never intended for anyone to hear any of it, but then my skills flourished here. Suddenly, I felt that I could participate. It didn’t matter whether it was good or bad, that was not where the focus was. The focus was on what it could do for others, what others could gain from it if I shared it. So, I have learned a lot about myself and my relationships with others through my participation in the MTW. I have gained confidence and grown as a person; from having a completely decimated self-worth to being confident enough to start searching, testing myself, trying and failing.

I have always felt like something was wrong with me and like everyone else was so different from me. Well, in fact, I have thought of myself as being “no-one.” One of my texts became a play in the MTW. It was based on my whole life, my entire sense of identity. It was about being “no-one,” and feeling left out and fighting alone against the world.

I had to think a little bigger than I normally do. I couldn’t sit there all narrow-minded and think that I was no-one. I had to think differently and get “no-one” to interact with the others. That was when I realized a lot about myself, that I can interact with others, even if I am a “no-one.” But that would mean that I wasn’t so alone anyway? Maybe I was “someone!”

### Analysis

Mina has always considered herself as a “no-one” and felt very much alone in the world. Through her descriptions, we see that there has been little room for self-exploration since being no one is such a constant. She narrates that there is no-one to explore. Her feeling of being different from others relates to her finding a place in society. Being a no-one is a strong self-critical voice. In the MTW, she benefitted from new experiences when she interacted with other people. For her it meant becoming part of a community and that focus was changed towards the product—the performance—instead of on her individual skills. In this way, her self-critical voice became diminished. Mina’s experience of being a part of a creative process enabled her to narrate a coherent story to an audience (Thomson & Jaque, 2017). By doing so, she became part of something bigger. The musicians and actors collaborated in an effort to realize a performance that was compelling and meaningful for the musicians, actors, and their audience, as Thomson and Jaque (2017) acknowledged. The fact that the participants could freely choose how to tell their story challenged Mina to see various texts and portray characters from different perspectives. She learned to open up to new experiences and interpretations.

The MTW enabled Mina to adjust her perception of reality by challenging her to let go of some of her negative beliefs and ways of thinking. By experimenting with the creative activities, she was able to entertain and embrace new, more positive ways of being (Rogers, 1967). As an actor in the MTW, she accepted a range of roles; the similarities these roles had to her own life appear to have enabled her self-exploration. Being able to portray the character “no-one” had a crucial impact on her new way of seeing herself. However, what was significant was the opportunity to bring life to the character of “no-one” on stage where “no-one” could interact with the other characters in the play. She had to use herself as a tool to allow the character to genuinely come to life for an audience (Hagen, 1973). In this process of portraying the character, she went beyond her old perceptions of herself and through a self-discovery journey from being “no-one” to becoming “someone.” Consistent with the findings from Tust-Gunn (1995), Mina experienced a dynamic interaction between understanding herself and exploring the character “no-one”; through this explorative process, Mina became familiar with undiscovered facets of her life and experienced a new understanding of herself. Mina experienced herself through becoming creative and the creative process enabled a deeper engagement with the self and the world around her (Nelson & Rawlings, 2009). Through participatory arts, she told the story of her self-exploration that went from a cognitive level to an emotional level. She increased her ability to challenge existing preconceived beliefs and she became open to new understandings about herself and others.

## Discussion and concluding remarks

The analyses of the narratives show that participation in the MTW gave participants new experiences and possibilities to change previous identities. Participation also contributed to processes that reflected both internal individual and personal identities and collective and socially created identities. Participants described an extended picture of themselves, incorporating a change of identity, from their previous stigmatized identity as being mentally ill to a more positive, reconstructed identity. In the two-phase process of portraying the characters (Noice & Noice, 2002), they each experienced a journey of self-discovery. They were able to use themselves as tools when they explored the script, determined the intentions of the characters and rehearsed and performed their roles. Their perceived identities and self-knowledge were the main sources drawn from to portray the characters. This required actors and musicians who were curious and wanted to accept and understand the different facets of themselves (Hagen, 1973). In line with Hagen’s understanding, the participants in the MTW learned more about themselves, which included the opportunity to explore their own sense of identity, to enlarge that sense of self and to see how they could utilize that knowledge when they portrayed a character (Hagen, 1973). As an alternative to therapy, the participants had the opportunity to work on themselves, exploring the questions “Who am I?” and “Who can I become?” In this explorative process, they were able to discover themselves in new ways, which could result in becoming familiar with undiscovered facets of their lives and sometimes new permanent understandings of themselves (Tust-Gunn, 1995).

The participants’ engagement in the MTW had a direct impact on their identities in relation to others in the workshops. The one unifying factor the participants shared when they commenced the MTW was their common experience of being labelled as having long-term mental health problems. However, after a while, their focus on arts became more important as they began to identify with the group. In everyday life, experiencing psychological belonging to a social group has been challenging for the participants (Hagerty, Lynch-Sauer, Patusky, Bouwsema, & Collier, 1992). Engaging in a creative environment where they felt accepted and staying together with people who shared their interests in music, theatre and scriptwriting gave them a sense of belonging, and for some of the participants, for the first time. Creating arts together gave the participants the experience of being part of a social world (Van Lith et al., 2009), which made it possible to question narratives about themselves and narratives regarding how society may look upon them. Through sharing and weaving together stories, anecdotes and narrative fragments from their lives, they collectively created counterstories (Nelson, 2001), extending their and the audience’s perspectives on mental health, the mental health system and being a human being. Consistent with findings from Stickley (2010), some of the participants experienced participation in music and theatre as an opportunity to collectively redefine themselves. It restored their identities as artists as a social response to their experience of having their identities spoiled by society.

For participants in this study, identity is not something solely within (Gergen, 2009); identities are fluid (Bauman, 2001) and dependent on others, their relations, and their contexts (Gergen, 2009). The participants recounted spending much time in mental health care, surrounded by other patients and health professionals. This engagement within the mental health discourse strongly influenced their personal and social identities. It is apparent that an illness identity endures; moreover, an identity as a mental health patient is doubly negative and is often internalized (spoiled) (Goffman (1968b). In this study, this spoiled identity is confronted by the possibility of identity transformation through experimental arts activities. Theatre and music facilitated different and new experiences contrary to the stigmatized or spoiled identities enforced by society and the mental health system. These findings can give insight into potential new understandings of the recovery processes. Bringing music and theatre in mental hospital contexts could help people to develop new, expanded and different narratives about themselves. Participants in this study formed new narratives, which in turn brought reflexivity in the way they understood and told stories about themselves. Neither the mental health problems, nor their identities of being an actor, musician or scriptwriter could provide the whole picture of them, just a part. They are in a process working on an identity narrative that they can live with and that tells themselves and the world around them about whom they are becoming as human beings. However, this does not imply that their previous identities are gone; their old identities remain and can be recalled in relation to themselves and others.

Deegan (1997) says, “recovery is a process, not an endpoint or a destination” (p. 20). Through the MTW, the hospital is working with identity as a key in people’s recovery processes (Andresen, Oades, & Caputi, 2003; Davidson & White, 2007; Salzmann-Erikson, 2013; Slade et al., 2012). As recovery is a personal and a social process (Topor, Borg, Di Girolamo, & Davidson, 2011), with no endpoint (Deegan, 1997), identity transformation can be the same. Based on the participants’ narratives, we can appreciate that identity work takes place not just internally and with the participants, but also in relation to others in other contexts. Understanding, redefining and accepting self; incorporating illness; and overcoming stigma are some of the essential building blocks of recovery (Davidson and White (2007). Participation in the MTW had the potential to influence all these elements of identity. We do not assert that participation in music and theatre necessarily changes people’s identities. However, this study illustrates how people with long-term mental health problems could work with their own identities in creative ways in the hospital context. Through participatory arts, people with long-term mental health problems have the potential to develop new identities and anchor points, which could result in expanded narratives of themselves.
